# Novel circular RNAs of the apoptosis‐related *BAX* and *BCL2L12* genes identified in a chronic lymphocytic leukemia cell line using nanopore sequencing

**DOI:** 10.1002/2211-5463.13672

**Published:** 2023-09-19

**Authors:** Christos K. Kontos, Paraskevi Karousi, Pinelopi I. Artemaki, Ahmed Abdelgawad, Aspasia Dimitriadou, Nikolaos P. Machairas, Diamantis C. Sideris, Vasiliki Pappa, Andreas Scorilas, Mona Batish, Sotirios G. Papageorgiou

**Affiliations:** ^1^ Department of Biochemistry and Molecular Biology, Faculty of Biology National and Kapodistrian University of Athens Greece; ^2^ Department of Medical and Molecular Sciences University of Delaware Newark DE USA; ^3^ Second Department of Internal Medicine and Research Unit University General Hospital “Attikon” Athens Greece

**Keywords:** BCL2 family, circRNA, pro‐apoptotic, single‐molecule fluorescence *in situ* hybridization, third generation (long‐read) sequencing, transcriptomics

## Abstract

Circular RNAs (circRNAs), a novel RNA type generated by back‐splicing, are key regulators of gene expression, with deregulated expression and established involvement in leukemia. The products of *BCL2* and its homologs, including *BAX* and *BCL2L12*, are implicated in chronic lymphocytic leukemia (CLL). However, to the best of our knowledge, nothing is known about circRNAs produced by these two genes and their role in CLL. We sought to further elucidate the contribution of *BAX* and *BCL2L12* in CLL by unraveling the identity, localization, and potential role of their circRNAs. Therefore, total RNA from the EHEB cell line and peripheral blood mononuclear cells (PBMCs) of CLL patients and non‐leukemic blood donors was extracted and reverse‐transcribed using random hexamers. Next, nested PCRs with divergent primers were performed and the purified PCR products were subjected to 3rd generation nanopore sequencing. Nested PCRs were also applied to first‐strand cDNAs synthesized from total RNA extracts of PBMCs from CLL patients and non‐leukemic blood donors. Lastly, a single‐molecule resolution fluorescent *in situ* hybridization method called circFISH was used to visualize the circRNA distribution in EHEB cells. We discovered several novel circRNAs produced by *BAX* and *BCL2L12*, which were characterized by great exon structure diversity. In addition, intriguing findings regarding their formation emerged. Interestingly, visualization of the most abundant circRNAs showed distinct intracellular localization. Moreover, a complex *BAX* and *BCL2L12* circRNA expression pattern was revealed in CLL patients and non‐leukemic blood donors. Our data suggest a multifaceted role of *BAX* and *BCL2L12* circRNAs in B‐cell CLL.

## Graphical abstract

Novel circular RNAs (circRNAs) deriving from *BAX* and *BCL2L12* were discovered using nanopore sequencing. These circRNAs exhibit diverse exon structures, distinct intracellular localization, and may interact with microRNAs with a proven role in chronic lymphocytic leukemia (CLL). A complex circRNA expression pattern in CLL patients and non‐leukemic blood donors was found as well. Our findings suggest a multifaceted role of these circRNAs in B‐cell CLL.

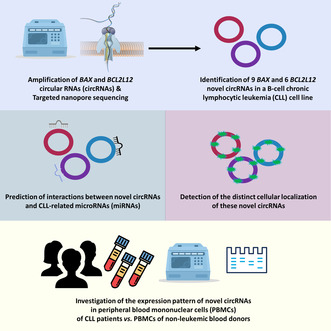

**Abbreviations**
NGS: next‐generation sequencing
circRNA: circular RNA
CLL: chronic lymphocytic leukemia
FISH: fluorescence *in situ* hybridization
miRNA: microRNA
RBP: RNA‐binding protein

Chronic lymphocytic leukemia (CLL) is the most common leukemia in adults. It is characterized by a typical defect in apoptosis and is still an incurable disease. Members of the BCL2 family, which are the key regulators of the mitochondrial apoptotic pathway, play a role in CLL pathogenesis [1]. The members of this family are grouped based on their function into pro‐ and anti‐apoptotic ones. Specifically, the balance between the pro‐ and anti‐apoptotic members of the BCL2 family is critical for the pathogenesis, chemorefractoriness, and clinical outcome of this disease [2].

The BAX/BCL2 ratio is a prognostic biomarker of particular significance for CLL. These molecules are two of the most well‐studied members of the BCL2 family. Specifically, *BAX* is a typical pro‐apoptotic member and acts in competition with the typical anti‐apoptotic member BCL2 [3]. Besides the aforementioned BCL2 family members, other members of the BCL2 family have been shown to play a pivotal role in CLL. Members of our research team have highlighted the clinical value of another BCL2 family member, *BCL2L12*, in CLL, suggesting its prognostic utility in this malignancy [4]. This gene exerts an anti‐apoptotic function, although its structure is not typical for an anti‐apoptotic BCL2 member. Since the pathogenesis of CLL has been linked to aberrant splicing, the alternative splicing of *BCL2* family members is quite an interesting research area. A typical example is *BCL2L1* (*BCLX*), a BCL2 family member with a proven role in CLL [5]; its alternative transcripts encode apoptosis‐related proteins with opposite roles.

Alternative splicing is a phenomenon observed not only in linear transcripts but also in circular ones, leading to the generation of multiple circular RNAs (circRNAs) from a single gene [6, 7]. CircRNAs are covalently closed, single‐stranded RNA molecules, generated from precursor mRNAs or tRNAs via the mechanism of back‐splicing. They were discovered in the ‘70s; however, they were characterized as byproducts of splicing. The advances in transcriptomics have brought them to the fore, and they have attracted researchers' interest due to their multifaceted roles in both physiological and pathological conditions. Specifically, circRNAs can act as microRNA (miRNA) sponges, protein scaffolds, and/or decoys, while there are indications for their translational potential and the transcriptional regulation of their parental genes. Moreover, they are implicated in the regulation of key signaling pathways and cellular processes [8, 9, 10, 11], including apoptosis. Therefore, the investigation of circRNAs deriving from apoptosis‐related genes is quite significant.

One of the most notable features of these molecules is their involvement in the development and progression of diseases, including hematological malignancies [9, 11]. Additionally, their prolonged half‐life time due to their resistance to exonucleolytic RNases render them ideal biomarker candidates. Their expression and role in leukemias have been investigated in several studies so far [12]. A characteristic example of circRNAs implicated in CLL development is circ_0132266, which interacts with miR‐337‐3p to modulate PML expression [13]. Moreover, Xia *et al*. [14] demonstrated that circ‐CBFB contributed to CLL progression by modulation of the miR‐607/FZD3/WNT/β‐catenin axis.

However, the knowledge regarding circRNAs in CLL, and particularly their alternative splice variants, is rather limited. Prompted by this, we decided to identify novel circRNAs of apoptosis‐related genes in CLL and investigate their back‐splicing pattern, focusing on the BCL2 family due to its key role in CLL pathogenesis. Our unpublished data support the intense alternative back‐splicing of *BCL2L12* in solid tumors. Taking into consideration this finding and the prognostic utility of linear *BCL2L12* transcripts in CLL [4], we decided to investigate the expression of *BCL2L12* circRNAs. Since BCL2L12 exerts an anti‐apoptotic function, we decided to investigate the circRNAs of a pro‐apoptotic BCL2 member as well (*BAX*). For this purpose, we conducted targeted 3rd generation sequencing based on nanopore technology, using the EHEB cell line. Moreover, we applied PCR assays to explore the expression pattern of the novel circRNAs in CLL patients' peripheral blood mononuclear cells (PBMCs). We identified 12 novel *BAX* circRNAs and 6 novel *BCL2L12* circRNAs with a potential role in CLL pathogenesis. Last, a complex *BAX* and *BCL2L12* circRNA expression pattern was revealed in CLL patients. Interestingly, the visualization of the most abundant of the novel circRNAs showed distinct cellular localization in EHEB cells.

## Methods

### Cell line culture

The human CLL cell line, EHEB, was purchased from the German Collection of Microorganisms (Braunschweig, Germany) and Cell Cultures (DSMZ) GmbH and cultivated. Cells were seeded at a density of about 0.5 × 10^6^ cells·mL^−1^ and incubated at 37 °C with 5% CO_2_, following the DSMZ guidelines.

### Sample collection and cell immunophenotyping

Peripheral blood mononuclear cells were collected from 24 CLL patients with an extremely high percentage of leukemic B‐cells in their blood and 16 non‐leukemic blood donors, using Ficoll–Hypaque gradient centrifugation. Cell immunophenotyping was performed as previously described [15]. The institutional Ethics Committee of the University General Hospital “Attikon” (Athens, Greece) approved this study, which was conducted in compliance with the Helsinki Declaration of 1975 (revised in 1983). Written informed consent was obtained from each study participant providing sample.

### Total RNA isolation and reverse transcription

According to the manufacturer's instructions, total RNA was extracted from the EHEB cell line and the human samples using the TRItidy G™ Reagent (AppliChem GmbH, Darmstadt, Germany), diluted in DEPC‐treated H_2_O and stored at −80 °C until further use. The concentration and purity of the total RNA extracts were evaluated spectrophotometrically at 260 and 280 nm, using the BioSpec‐nano Micro‐volume UV–Vis Spectrophotometer (Shimadzu, Kyoto, Japan). RNA integrity was assessed with agarose gel electrophoresis.

Next, 2 μg of total RNA from EHEB cells and 500 ng of total RNA from each patient's PBMC sample were reverse‐transcribed. Each reaction mixture also contained 50 ng of random hexamers, dNTP mix (0.5 mm each), and DEPC‐treated H_2_O, in a final volume of 10 μL. The mixture was heated to 65 °C for 5 min and then quickly chilled on ice. Next, the reaction mixture was adjusted to contain First‐Strand Buffer (1×), DTT (10 mm), 40 U RNaseOUT™ recombinant RNase inhibitor (Invitrogen™, Thermo Fisher Scientific Inc., Carlsbad, CA, USA), and 200 U MMLV reverse transcriptase (Invitrogen™), in a final reaction volume of 20 μL. The mixture was incubated at 25 °C for 10 min and then at 37 °C for 50 min, and finally, the reverse transcriptase was inactivated at 70 °C for 15 min. The first‐strand cDNA synthesis was performed in a MiniAmp Thermal Cycler (Applied Biosystems™, Thermo Fisher Scientific Inc., Waltham, MA, USA).

### Amplification of *BAX* and *BCL2L12* circRNAs in EHEB cells

Two pairs of divergent primers were designed on each coding exon of *BAX* and *BCL2L12*, so that only cDNAs having derived from circRNAs could be amplified during the nested PCR assays [16]. In cases where the exon length or GC content did not allow designing four different primers on the same exon, semi‐nested PCR was conducted after designing three primers on this exon. The sequences of the primers used for *BAX* and *BCL2L12* circRNAs are listed in Table S1. Each PCR reaction was conducted using KAPA Taq DNA Polymerase (KAPA Biosystems Inc., Woburn, MA, USA) in a MiniAmp Thermal Cycler (Applied Biosystems™, Thermo Fisher Scientific Inc.). For the amplification of *BAX* and *BCL2L12* circRNAs in EHEB cDNA, the reaction mix contained KAPA Taq Buffer (1×) including MgCl_2_ (1.5 mm), dNTP mix (0.2 mm each), each primer (400 nm), 1.25 U KAPA Taq DNA Polymerase, 0.5 μL of template (EHEB cDNA in the first‐round PCR; 1 : 50 diluted PCR product in the nested PCR), and DEPC‐treated H_2_O, in a final volume of 25 μL. The applied thermal protocol is the following one: an initial denaturation step at 95 °C for 3 min, followed by 25 or 30 cycles (for *BAX* and *BCL2L12*, respectively) of a denaturation step at 95 °C for 30 s, an annealing step for 30 s (annealing temperature for each primer pair is shown in Table S1), and an elongation step at 72 °C for 1 min; a final elongation step was carried out at 72 °C for 1 min. The PCR products were purified using spin columns.

### 3rd Generation sequencing using nanopore technology

The nested PCR products of each gene from the EHEB cDNA were mixed at equal volumes and purified using spin columns (Macherey‐Nagel GmbH & Co. KG, Düren, Germany). The concentration of the purified PCR product pools was determined using a Qubit^®^ 2.0 fluorometer (Thermo Fisher Scientific, Inc.). Both purified PCR product pools were subjected to 3rd generation sequencing. Briefly, the NEBNext^®^ Ultra™ II End Repair/dA‐Tailing Module (New England Biolabs Inc., Ipswich, MA, USA) was used for the end‐repair process, the Agencourt AMPure XP beads magnetic beads (Beckman Coulter, Brea, CA, USA) were used for the nucleic acid purification steps, while the Quick T4 Ligase (New England Biolabs Inc.) enabled the sequencing adapter ligation. As a result, two barcoded libraries corresponding to purified PCR products deriving from *BAX* circRNAs and *BCL2L12* circRNAs were constructed. The derived barcoded libraries were quantified using the Qubit^®^ 2.0 fluorometer and equimolarly mixed. Then, nanopore sequencing was carried out using the Flongle adaptor on the MinION Mk1C sequencer (Oxford Nanopore Technologies Ltd., Oxford, UK). A FLO‐FLG001 flow cell with R9.4.1 chemistry, the Ligation Sequencing Kit, and the Native Barcoding Expansion 1–12 were used, following the manufacturer's instructions.

### circRNA annotation

During the sequencing run, 22 801 reads were generated for *BAX* and 26 170 reads for *BCL2L12*. The primary analysis of the acquired nanopore sequencing data including basecalling, demultiplexing, adapter trimming, and quality assessment was performed with guppy [17]. The obtained nanopore sequencing raw data were mapped to chromosome 19 of the human reference genome (hg38) with the minimap2 aligner [18]. Mapped sequencing reads were visualized with Integrative Genomics Viewer (igv) [19], aiming to identify the single‐exon circRNAs.

For the identification of multi‐exon circRNAs, we implemented our *in‐house* developed algorithm, “ASDT” [20]. ASDT is a generic splicing tool capable of identifying alternative splicing events from high‐throughput sequencing datasets. However, circRNAs possess a unique back‐splice site that is absent from the linear transcripts. For this reason, we appropriately modified the complete records of both genes with “.gb” extension from the NCBI Reference Sequence Database (RefSeq); the modified files served as input for our algorithm, so that the sequencing reads covering circRNAs with high accuracy could be automatically detected using the ASDT tool. More specifically, 5 and 7 modified “.gb” files were constructed for *BAX* and *BCL2L12*, respectively. Each file was modified so that the first and the last exon in the gene sequence were identical. The number of generated “.gb” files was the same as the number of the different PCR assays conducted for each gene, namely 5 for *BAX* and 7 for *BCL2L12*. Next, we used our *in‐house*–developed, publicly available algorithms, namely ASDT remodeler (https://github.com/pkarousi/ASDT_remodeler) and Read catcher (https://github.com/pkarousi/Read_catcher), for the detection of multi‐exon circRNAs.

For the comparison of novel circRNAs with linear *BAX* and *BCL2L12* transcripts, an RNA‐seq dataset (ID: SRX6608451), derived from next‐generation sequencing (NGS) in total RNA extracted from EHEB cells, was downloaded from the Sequence Read Archive (SRA) of NCBI and mapped to the human reference genome (hg38). The coverage of the intronic and exonic regions of *BAX* and *BCL2L12* was determined using the samtools coverage module.

### Sanger sequencing of the amplicons deriving from particular *BAX* and *BCL2L12* circRNAs

Amplicons generated using nested PCR for the most frequently detected *BCL2L12* circRNA (circ‐BCL2L12‐48) and one of the two most frequently detected *BAX* circRNAs (circ‐BAX‐4) were also subjected to Sanger sequencing, so that each back‐splice junction sequence be further verified. After specifically amplifying the circRNAs of interest using PCR thermal protocols similar to the aforementioned (primer pairs are shown in Table S2), the nested PCR products were electrophoresed on a 3% agarose gel, the specific bands were properly excised from the gel and gel‐extracted using a Gel and PCR Clean‐Up kit (Macherey‐Nagel GmbH and Co. KG). The concentration of each purified nested PCR product was determined using Qubit^®^ 2.0 fluorometer. For amplicon sequence verification, Sanger sequencing was carried out in both directions, starting from each nested PCR primer (Table S2).

### circFISH imaging

A set of probes was designed to target the regions of *BAX* and *BCL2L12* found in each respective circRNA (Table S3), as previously described [21]. Briefly, the probes were synthesized with a terminal ‐NH_2_ modification by LGC Biosearch Technologies Inc (Berlin, Germany). These probes were pooled and labeled in mass with Texas red fluorophore. The labeled fraction was purified using reverse‐phase HPLC. EHEB cells were fixed, permeabilized, and hybridized with the labeled probes. The next day, unbound probes were washed off and the cells were mounted with DAPI‐containing mounting medium and imaged using 100× oil objective in a Nikon Eclipse Ti‐E Inverted Epifluorescence Microscope (Nikon Corporation, Minato City, Tokyo, Japan) equipped with a CCD Princeton Pixis 1024b camera. The images were acquired using metamorph software and analyzed using a custom‐written program for spot counting in matlab (Mathworks Inc, Natick, MA, USA).

### Expression analysis of *BAX* and *BCL2L12* circRNAs in CLL and normal PBMC samples

For the amplification of *BAX* and *BCL2L12* cDNAs having derived from circRNAs in PBMC samples, the reaction mix contained KAPA Taq Buffer (1×) including MgCl_2_ (1.5 mm), dNTP mix (0.2 mm each), each primer (400 nm), 1.25 U KAPA Taq DNA Polymerase, 1.0 μL of template (1 : 6 diluted cDNA in the first‐round PCR; 1 : 50 diluted PCR product in the nested PCR), and DEPC‐treated H_2_O, in a final volume of 25 μL. The applied thermal protocol is the following one: an initial denaturation step at 95 °C for 3 min, followed by 30 cycles (for both *BAX* and *BCL2L12*) of a denaturation step at 95 °C for 30 s, an annealing step for 30 s (annealing temperature for each primer pair is shown in Table S4), and an elongation step at 72 °C for 1 min; a final elongation step was carried out at 72 °C for 1 min. The nested PCR assays in cDNAs from CLL and normal PBMC samples were conducted only with the primers annealing in exon 3 of both *BAX* and *BCL2L12*; this selection was based on the fact that this exon was found to be present in the majority of the novel circRNAs identified in the EHEB cell line. The nested PCR products were electrophoresed on 2% agarose gels, next to 50 bp DNA ladder (New England Biolabs Inc.).

### Bioinformatic analysis

We investigated whether circRNAs of *BAX* and *BCL2L12* had already been submitted in the publicly available databases: CircAtlas 2.0 [22], CIRCpedia v.2 [23], circBase [24], and LeukemiaDB [25]. All of the novel circRNAs were analyzed regarding their ability to sponge miRNAs, via the custom prediction tool of the miRDB database [20]. More specifically, each circRNA sequence was submitted to the Custom Prediction tool of miRDB as a target sequence; in this way, all miRNAs that possibly bind to each circRNA of interest are obtained along with a score indicating the probability of binding. Next, all the miRNAs predicted to be sponged by a novel circRNA were evaluated in the dbDEMC (database of Differentially Expressed MiRNAs in human Cancers) database regarding their expression levels in CLL [26]. Finally, one of the most frequently detected *BAX* circRNA was analyzed regarding its ability to interact with proteins. CLIP‐seq data from the ENCORI database were analyzed for this purpose [27].

## Results

### Coverage of the *BAX* and *BCL2L12* genes by circRNAs

**Fig. 1:**
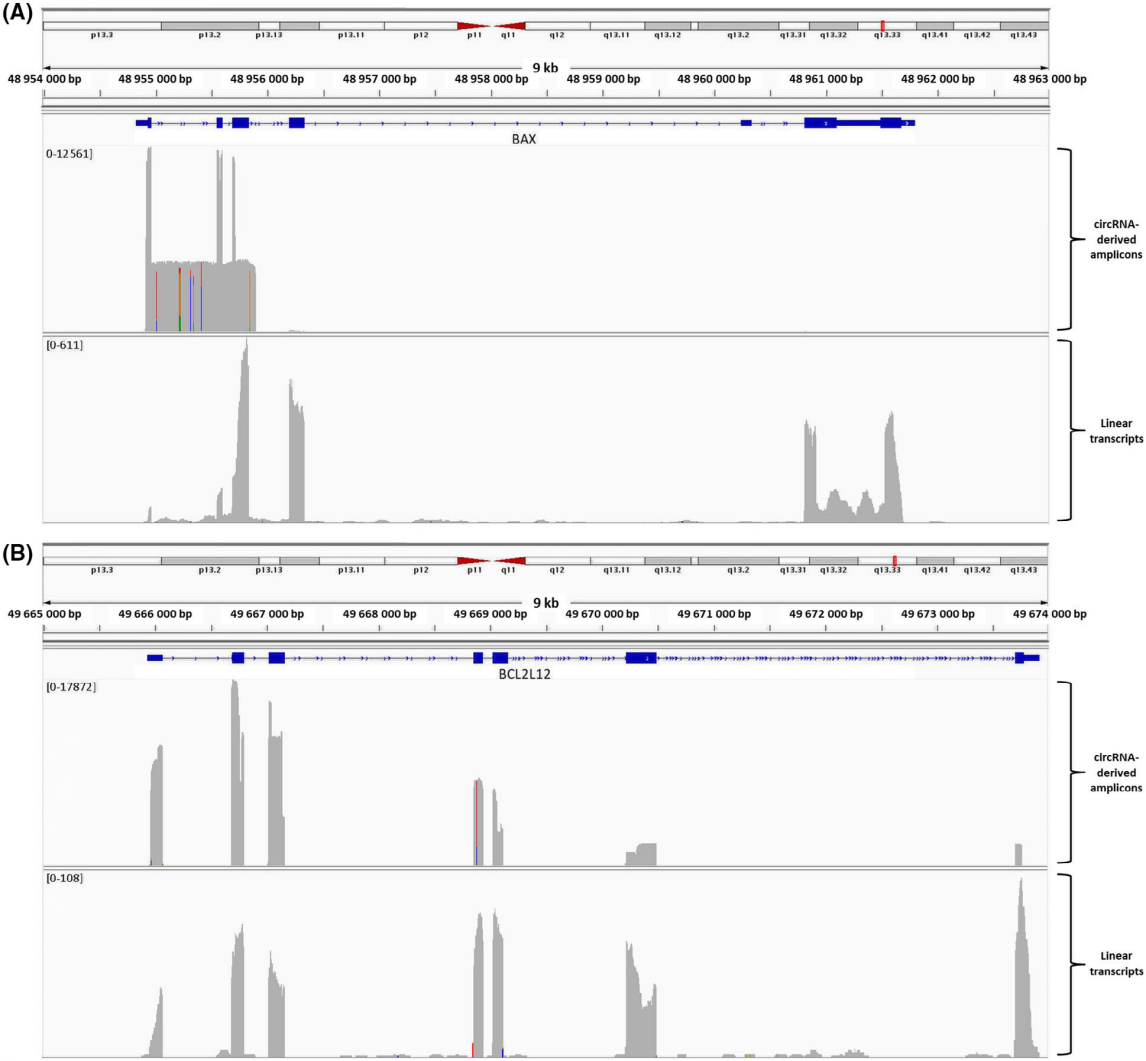
Visualization of the coverage of *BAX* (A) and *BCL2L12* (B) genomic sequences by our full‐length reads of circRNA‐derived amplicons (obtained using nanopore sequencing) and by RNA‐seq reads from a publicly available dataset (based on total RNA from EHEB cells), using the Integrative Genomics Viewer (igv). The scale of the vertical axis in all plots is linear.

The coverage of *BAX* genomic region by the *BAX* circRNA‐derived amplicons was remarkably different from the respective coverage by linear *BAX* transcripts (Fig. 1A). In more detail, BAX circRNAs covered cumulatively the three first exons of this gene as well as the intervening introns; the intron between exons 3 and 4 was slightly covered, too (Fig. S1A). In contrast, linear transcripts covered mostly exons 2, 4, 6, and 7. Additionally, the intron between *BAX* exons 6 and 7 was covered in the RNA‐seq dataset; low coverage of the intron between *BAX* exons 2 and 3 was also observed, yet at a much lower extent (Fig. S1A). At this point, it should be noted that exons 6 and 7 are also covered by *BAX* circRNA‐derived amplicons, yet this finding can only be visualized in IGV plots with a vertical axis log_10_ scale.

On the other hand, the *BCL2L12* circRNA‐derived amplicons covered cumulatively all seven exons of this gene, similarly to its linear transcripts (Fig. 1B). However, *BCL2L12* exons 6 and 7 were significantly under‐represented in circular transcripts, in comparison with the linear ones (Fig. S1B). Moreover, some introns of this gene showed a slight coverage by linear transcripts; however, since these reads were scattered in the introns (Fig. 1B, left panel), this alignment could be faulty due to the short length of NGS reads.

### Identification of novel *BAX* and *BCL2L12* circRNAs

**Fig. 2:**
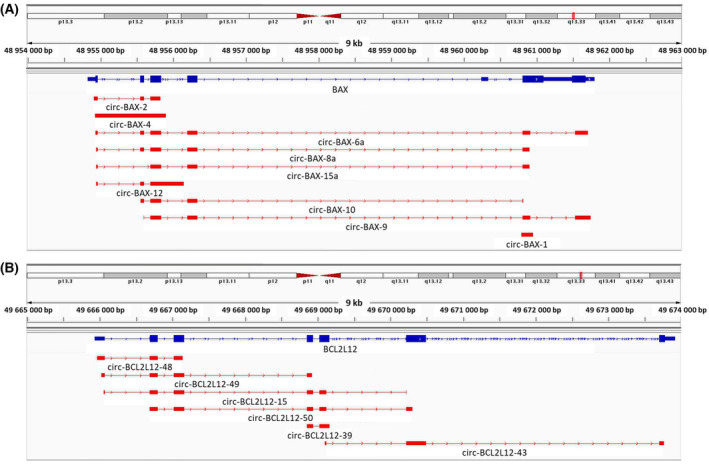
Visualization of the alignment of the novel *BAX* (A) and *BCL2L12* (B) circRNAs identified in EHEB cells, using the Integrative Genomics Viewer (igv). Each circRNA is aligned against the respective genomic sequence. All circRNAs are depicted starting from the back‐splice acceptor site.

Through the bioinformatic analysis of our experimental nanopore sequencing datasets, nine *BAX* circRNAs (Fig. 2A) and six *BCL2L12* circRNAs (Fig. 2B) were identified in the ΕΗΕΒ cell line. By analyzing public databases, we found that five *BAX* and four *BCL2L12* circRNAs are deposited in CIRCpedia v.2.0, 2 *BAX* and three *BCL2L12* circRNAs in CircAtlas 2.0, five *BAX* and five *BCL2L12* circRNAs in circBase, and two *BAX* and three *BCL2L12* circRNAs in LeukemiaDB. Most of these circRNAs are common between the publicly available databases. Out of the 18 circRNAs that were experimentally identified in our research study, only circ‐BCL2L12‐39 has previously been deposited to CircAtlas 2.0 and LeukemiaDB; all other circRNAs discovered via our novel approach are described for the first time. The sequences of the nine *BAX* circRNAs and six *BCL2L12* circRNAs have been deposited in GenBank^®^ (Table 1).

**Table 1:** The novel *BAX* and *BCL2L12* circRNAs that were identified in the EHEB cell line.

Gene	circRNA ID	GenBank^®^ accession no.
*BAX*	circ‐BAX‐1	OQ139910.1
	circ‐BAX‐2	OQ139911.1
	circ‐BAX‐4	OQ139912.1
	circ‐BAX‐6a	OQ139913.1
	circ‐BAX‐8a	OQ139914.1
	circ‐BAX‐9	OQ139915.1
	circ‐BAX‐10	OQ139916.1
	circ‐BAX‐12	OQ139917.1
	circ‐BAX‐15a	OQ139918.1
*BCL2L12*	circ‐BCL2L12‐15	ON141947.1
	circ‐BCL2L12‐39	ON141971.1
	circ‐BCL2L12‐43	ON141975.1
	circ‐BCL2L12‐48	ON746137.1
	circ‐BCL2L12‐49	ON746138.1
	circ‐BCL2L12‐50	ON746139.1

The detected circRNAs of both genes were characterized by remarkable diversity in their structure. Only one *BAX* circRNA (circ‐BAX‐4) consisted of a single exon, whereas all other discovered *BAX* and *BCL2L12* in EHEB cells were composed of multiple exons. Moreover, only two *BAX* circRNAs spanned both exonic and intronic sequences of this gene, with circ‐BAX‐4 being one of them. Additionally, 6 out of 9 *BAX* circRNAs and 3 out of 6 *BCL2L12* included the first exon of the respective gene. It should be noted that exclusively intronic circRNAs could not have been amplified and identified, due to the fact that all primers were designed to anneal to exons. Last, circ‐BAX‐4 and circ‐BCL2L12‐48 were the most frequently detected circRNAs in our datasets.

### Features of the back‐splice junctions of the novel circRNAs

All “forward” splice junctions detected in the 15 circRNAs are encountered in linear transcripts of *BAX* and *BCL2L12* genes, as consecutive exons are spliced together; in only one case (circ‐BAX‐15a), *BAX* exon 2 was skipped (Fig. 2B), but this also a feature of a *BAX* mRNA resulting from alternative splicing of the pre‐mRNA. On the other hand, back‐splice junctions were mostly formed between non‐canonical splice sites residing within coding exons or – in the two cases of exonic–intronic circRNAs (circ‐BAX‐4 and circ‐BAX‐12) – within introns (Fig. 2B). Interestingly, only one back‐splice junction was formed by two full‐length exons (Fig. 2A); thus, circ‐BCL2L12‐39 results from the back‐splicing of the 3′‐extended exon 5 (141 nt) of *BCL2L12* to exon 4 (87 nt). However, the circRNA sequences currently deposited in the aforementioned public databases are characterized only by canonical splice sites in their back‐splice junction.

**Fig. 3:**
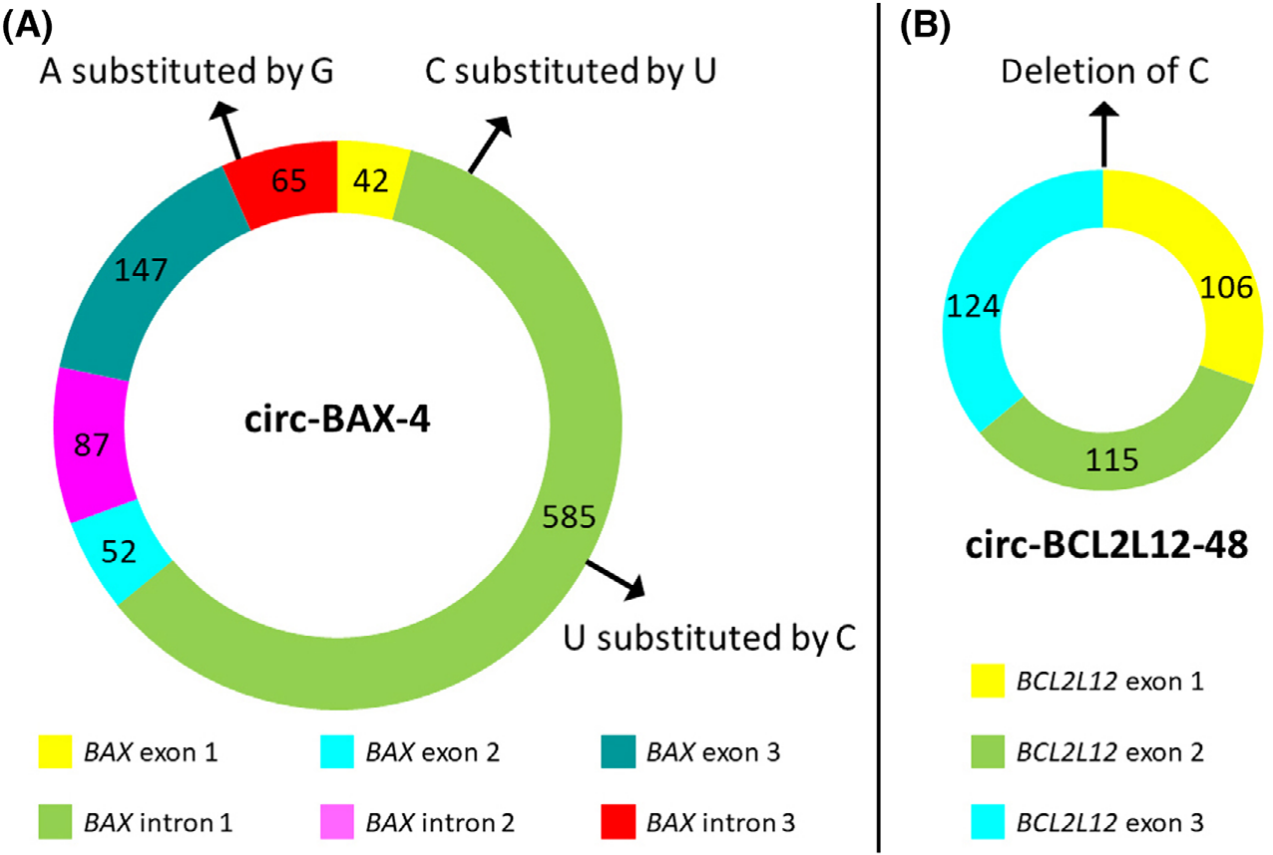
Schematic representation of circ‐BAX‐4 (A) and circ‐BCL2L12‐48 (B), the most represented circRNAs in our nanopore sequencing datasets. The single nucleotide variants that were found in each of the two sequences are indicated with arrows.

Another interesting finding was the existence of single nucleotide variants, compared to the reference sequence. Specifically, three altered nucleotides were observed at particular positions in the amplicon resulting from circ‐BAX‐4 cDNA amplification. These variants reflect base substitutions in the sequence of this circRNA (Fig. 3A). Regarding circ‐BCL2L12‐48, a deletion of a cytidine (C) nucleotide was observed in the sequence of the back‐splice junction; this variation revealed by nanopore sequencing was confirmed by Sanger sequencing (Fig. 3B).

### Intracellular localization of the novel circRNAs

**Fig. 4:**
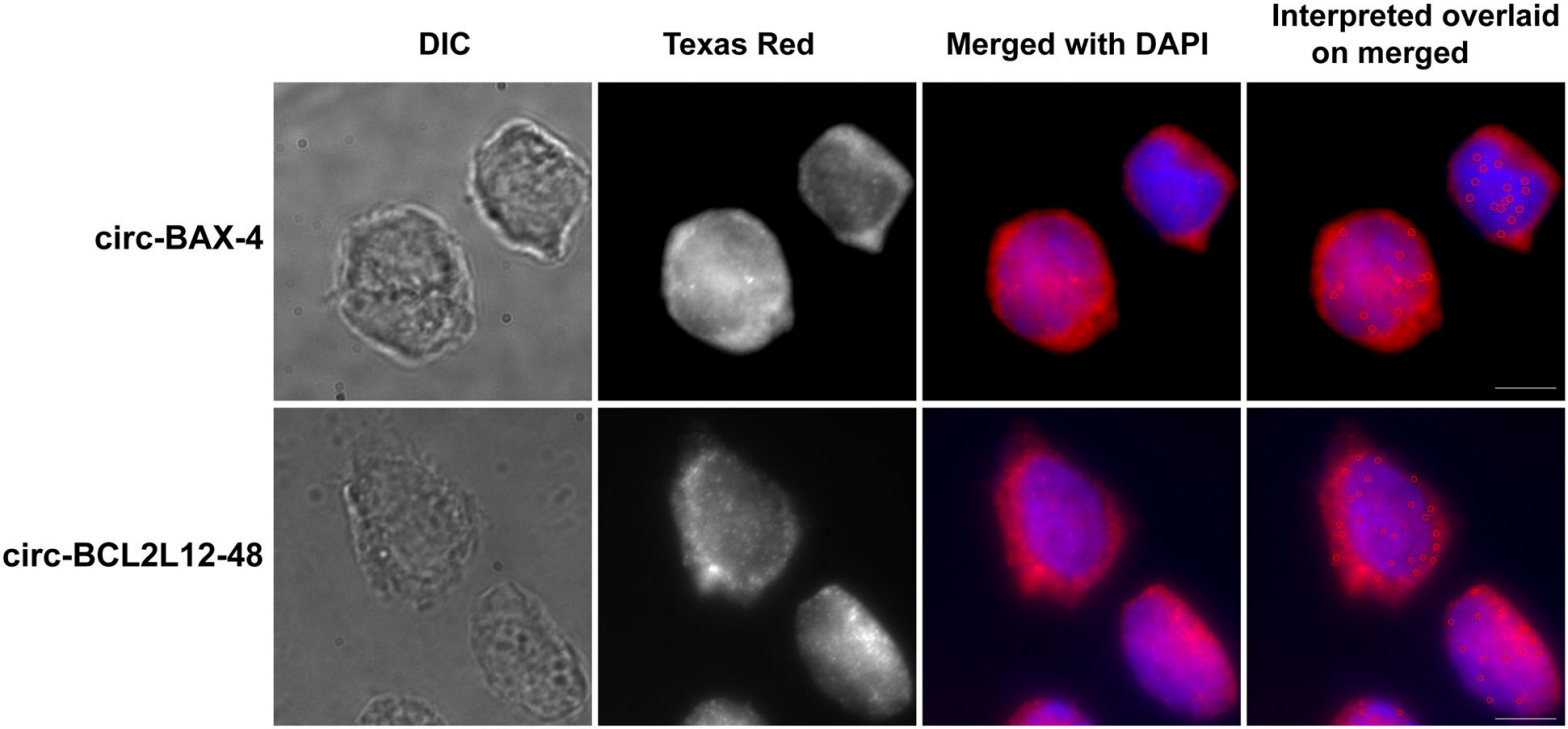
Visualization of the cellular localization of circ‐BAX‐4 and circ‐BCL2L12‐48 using circFISH: the EHEB cells were imaged using probes specific for regions that are present in circRNAs. The 1st panel from the left side is composed of the bright field images; the next panel represents a merged z stack of the raw signal observed in the Texas red channel (the fluorophore of the probes); the 3rd panel shows the merge of the nucleus stained with DAPI pseudo‐colored as blue and the circRNA signal pseudo‐colored as red. The last panel shows the merged image overlaid, with the interpreted output of matlab analysis with open red circles highlighting the circRNA signal. All images were obtained at 100× resolution. The scale bar is 5 μm.

Since circRNA function is largely determined by their localization in the cell, we utilized a single‐molecule fluorescence *in situ* hybridization (smFISH) method, called circFISH [22], to determine the cellular localization of the most abundant circRNA produced by each of the two genes. We designed probe sets that specifically bind the regions of the genes found in the two selected circRNAs and not in linear transcripts of these genes. Upon analysis, we found that both circ‐BAX‐4 and circ‐BCL2L12‐48 are present in both the cytoplasm and nucleus. Interestingly, the signal for circ‐BAX‐4 was mainly accumulated in the nucleus, while circ‐BCL2L12‐48 was found equally distributed in both compartments (Fig. 4). The observed intracellular distribution indicates that circ‐BAX‐4 is potentially involved in regulating transcription, while circ‐BCL2L12‐48 is more likely to exert a miRNA‐ and/or RNA‐binding protein (RBP)‐sponging function.

### Putative interactions of the novel circRNAs

Due to the high frequency of circ‐BAX‐4 in our dataset, its intronic regions were analyzed – based on CLIP‐seq experiments – regarding their ability to bind RBPs. This analysis revealed several potential interactions with splicing factors and RBPs (Table 2). Based on the miRDB prediction tool, all novel circRNAs are predicted to interact with miRNAs (Table S5). Downstream analysis of the miRNAs which are predicted to be sponged by the novel circRNAs in the dbDEMC database revealed which of these miRNAs show deregulated expression in CLL patients (Table 3).

**Table 2:** The proteins that are predicted to bind to the intronic regions of circ‐BAX‐4 and their respective roles, according to the analysis of CLIP‐seq data from the ENCORI database.

Protein	Role
RBFOX2	Alternative splicing regulation
SRSF1	Alternative splicing regulation
HNRNPU	Alternative splicing regulation
U2AF1	Alternative splicing regulation
HNRPA1	Alternative splicing regulation
SRSF3	Alternative splicing regulation
FMR1	Alternative splicing regulation and stability
DDX42	ATP‐dependent RNA helicase
HNRNPM	Binds avidly to poly(G) and poly(U) RNA homopolymers
DDX54	Inhibitor of transcription
SLTM	Inhibitor of transcription
DGCR8	miRNA biogenesis
CSTF2T	mRNA polyadenylation
IGF2BP2	mRNA transport regulation
HNRNPK	Nuclear metabolism
HNRNPA2B1	Nuclear metabolism
PRPF8	Pre‐mRNA splicing
SRSF7	Pre‐mRNA splicing
EIF4A3	Pre‐mRNA splicing
NONO	Pre‐mRNA splicing
U2AF2	Pre‐mRNA splicing and 3′‐end processing
FTO	RNA oxidative demethylation
ELAV1	Stability
GTF2F1	Transcription regulation
ZNF184	Transcription regulation
HNRNPUL1	Transcription regulation
IGF2BP3	Transportation

**Table 3:** circRNAs and miRNAs that are predicted to be sponged, with concomitant deregulated expression levels in CLL patients, compared to normal individuals.

Gene	circRNA	Targeted miRNA^a^	miRNA expression in CLL patients vs. normal controls^b^
*BAX*	circ‐BAX‐4	miR‐378a‐3p	Lower
*BCL2L12*	circ‐BCL2L12‐43	miR‐455‐3p	Lower
	circ‐BCL2L12‐48	miR‐181a‐2‐3p	Lower

^a^ Prediction derived from the miRDB tool.

^b^ Data derived from the dbDEMC 2.0 database.

### Detection of novel *BAX* and *BCL2L12* circRNAs in CLL patient samples

**Fig. 5:**
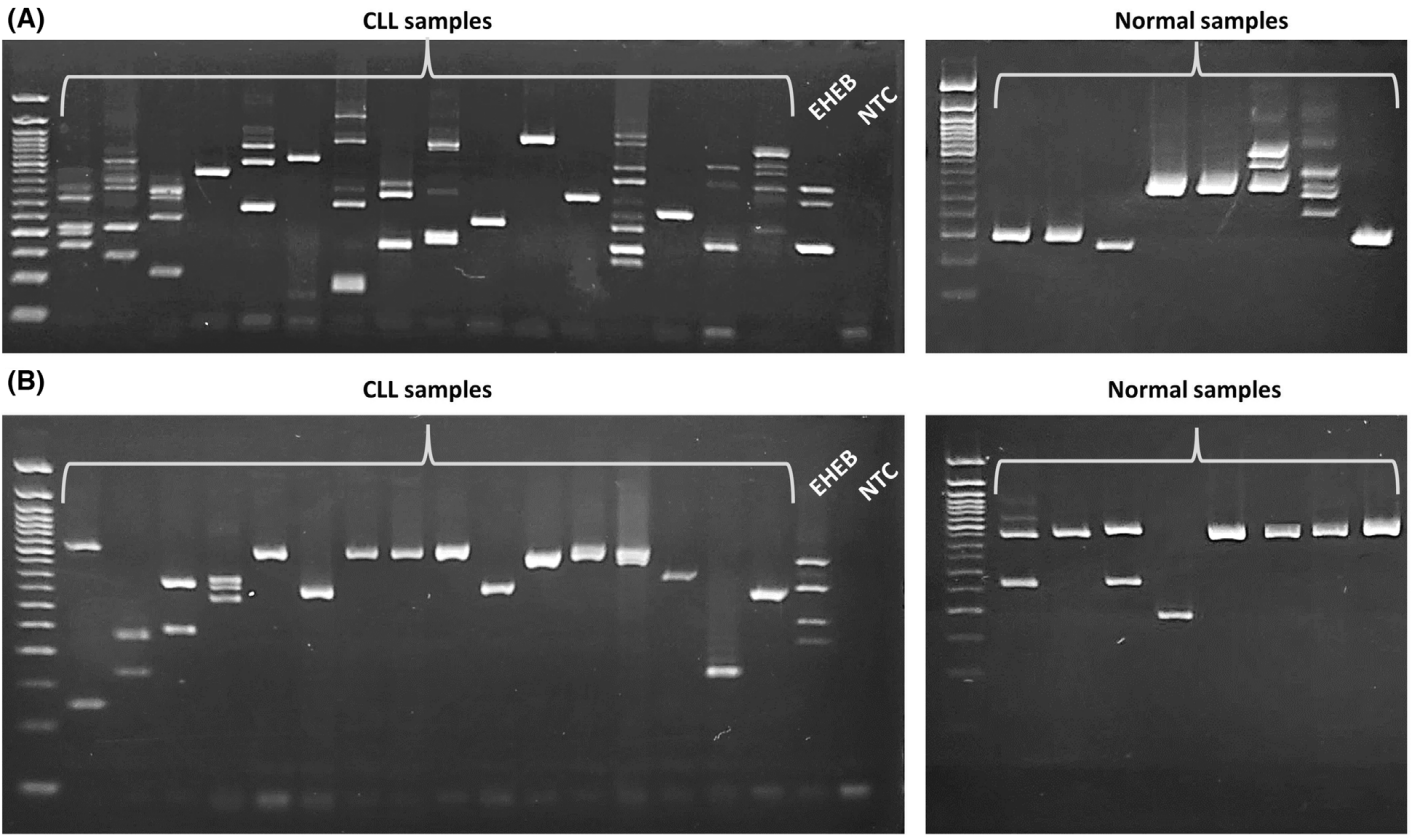
Agarose gel electrophoresis of the PCR products after amplification of *BAX* (A) and *BCL2L12* (B) circRNAs in CLL samples and normal controls, as well as in the EHEB cell line. DNA ladder: 50 bp; NTC, non‐template control.

Expression analysis of *BAX* and *BCL2L12* circRNAs in cDNAs derived from CLL patients' and non‐leukemic blood donors' PBMC samples was attempted with the use of nested PCR with divergent primers annealing to exon 3 of each gene, as described in detail in the Methods section. Interestingly, the circRNA expression pattern of both genes differs significantly among all CLL patient samples. Although some of the circRNAs identified in the EHEB cell line were detected in the human samples as well, we have also observed specific amplicons not corresponding to those discovered in EHEB cells. A representative example of this expression analysis in 16 CLL and 8 normal PBMC samples is presented in Fig. 5.

## Discussion

The advances in transcriptomics have revolutionized our knowledge regarding RNA biology by revealing several unknown aspects in this field. The discovery of circRNAs represents one such example. Despite the progress that has been achieved in this field, there are several unanswered questions regarding their biogenesis and function. One phenomenon regarding circRNA biogenesis that has attracted researchers' interest is the alternative back‐splicing, since it further expands the complexity of circRNA formation. However, alternative back‐splicing as a process remains largely unknown [6].

The poor investigation of this phenomenon is mainly attributed to the fact that research efforts have focused so far on the identification of circRNAs, and most of the available technology has not allowed for high sequencing depth. In particular, NGS is the most common technology that is used for this purpose; however, it is based on the detection of only the back‐splice site for the identification of each circRNA, and hence, the whole sequence of each circRNA can only be presumed [28, 29]. On the contrary, 3rd generation sequencing lifts this limitation by revealing the full sequence of molecules. Rahimi *et al*. [30] performed a global examination of full‐length circRNAs in human and mouse brains; however, a step of fragmentation was included in the library construction, leading to the necessity of read assembly. Aiming to avoid the fragmentation of the circRNAs, we implemented a PCR‐based approach followed by 3rd generation nanopore sequencing. Since the mechanism of circRNA production by back‐splicing is an active area of research, the deep investigation of the circRNAs of specific genes could assist in the better understanding of circRNA biology [6]. Prompted by these, we sought to investigate the alternative circRNAs of the apoptosis‐related genes, *BAX* and *BCL2L12* in CLL.

This high‐throughput approach led to the discovery of 12 *BAX* circRNAs and 6 *BCL2L12* circRNAs in CLL. The deep analysis of the circRNAs of these two genes revealed some common characteristics between them. Specifically, most circRNAs of both genes comprise multiple exons (at least 3). However, the current literature supports that most circRNAs consist of 2 or 3 exons [31]. Interestingly, the sequences of circ‐BAX‐4 and circ‐BCL2L12‐48, which are among the most frequent circRNAs detected in our CLL cell line, were characterized by some sequence variations, compared to the reference sequence. In circ‐BAX‐4, 3 nucleotide substitutions were detected in introns 1 and 3. In circ‐BCL2L12‐48, a deletion of a C in exon 1 was detected. These inconsistencies compared to genomic sequences have also been validated with Sanger sequencing; therefore, they cannot be attributed to errors during nanopore sequencing. Interestingly, nucleotide deletion has not been detected as an RNA modification in the current literature. To the best of our knowledge, the substitution of C by U could derive from the deamination of C, while the substitution of A by G could result from A‐to‐I editing since inosine (I) could be resolved as guanosine upon sequencing. A‐to‐I editing is an important regulatory mechanism that affects the stability of RNA and its interactions with other molecules, while it has also been associated with circRNA biogenesis. Since RNA base modifications, particularly in circRNAs, constitute a hot research topic and have been implicated in CLL and cancer pathogenesis in general [32, 33, 34], the aforementioned variations in the circRNA sequences merit further investigation and could add novel insights regarding the biogenesis of circRNAs.

Another novel finding of the present study refers to the back‐splice sites of the identified circRNAs. Most known circRNAs derive from internal exons of protein‐coding genes and require both 5′ and 3′ splice sites to be canonical for the formation of their back‐splice junction [9, 35]. However, the findings of the present study support the existence of circRNAs with non‐canonical splice sites forming the back‐splice junction; these circRNAs seem to constitute the majority in our dataset. Moreover, most of these non‐canonical back‐splice sites reside within coding exons of *BAX* or *BCL2L12*. An independent study that identified circRNAs deriving from the mouse *ApoA4* gene supports our findings regarding non‐canonical splice sites in the back‐splice junction formation. Specifically, analysis of the top 200 mouse *ApoA4* back‐splice junctions revealed the lack of canonical acceptor and donor splice sites, which emphasizes the complexity of circRNA formation [36]. We hypothesize that RBPs that are not considered as classical splicing factors may be involved in back‐splicing.

Despite the several common characteristics of *BAX* and *BCL2L12* circRNAs, there is quite a significant difference in their structures. Interestingly, one of the two most frequently detected *BAX* circRNAs fully retains introns 1 and 2, and partly intron 3. However, none of the exonic–intronic *BCL2L12* circRNAs had a high frequency compared to the exonic ones. So far, the most frequently detected and studied circRNAs were exonic; therefore, the discovery of an exonic–intronic circRNA in such an abundance is quite interesting and raises questions regarding its function. Since these intronic regions are rarely included in linear transcripts, a competition between forward‐ and back‐splicing as well as a functionality of the intronic regions could be proposed [8, 37, 38]. Moreover, circRNAs with both exonic and intronic regions are predicted to interact with RBPs and affect the transcription of their linear counterparts [39, 40, 41]. The publicly available CLIP‐seq data support the binding of splicing factors in these intronic regions, supporting a potential impact of these circRNAs on transcription and splicing [27].

*BAX* and *BCL2L12* play a significant role in CLL pathogenesis and several questions remain unanswered regarding defective apoptosis in CLL. Based on these, the discovery of plenty of circRNAs enhances the regulatory complexity. Therefore, it is intriguing to investigate the potential role of these novel circRNAs and, in particular, their miRNA sponging potential, since it is one of the most well‐known functions of circRNAs [10, 42]. Interestingly, *BCL2L12* circRNAs are predicted to sponge miR‐455‐3p, the expression levels of which are downregulated in CLL patients, compared to normal controls. Additionally, this miRNA has been characterized as a tumor suppressor in several malignancies [43, 44]. Furthermore, circ‐BCL2L12‐48, the most frequently detected *BCL2L12* circRNA in EHEB cells, is predicted to sponge miR‐181a‐2‐3p, the expression levels of which are downregulated in CLL patients, compared to normal individuals. Moreover, this miRNA is predicted to bind to *FLI1* mRNA, probably leading to a decrease in its expression. FLI1 is a transcription factor exhibiting high expression in leukemias, while its knockdown was shown to significantly hinder cell proliferation of leukemic cell lines [45]. Therefore, a potential regulatory axis consisting of circ‐BCL2L12‐48/miR‐181a‐2‐3p/*FLI1* could emerge in CLL.

Moreover, the two most frequently detected *BAX* circRNAs in EHEB are predicted to sponge miR‐296‐5p. This miRNA has been characterized as a tumor suppressor in several malignancies and its upregulation can induce apoptosis [46, 47, 48]. Last but not least, circ‐BAX‐4 is predicted to sponge miR‐378a‐3p, the expression levels of which are lower in CLL patients, compared to normal individuals. According to miRTarBase, miR‐378a‐3p binds to *VEGFA* mRNA, which is upregulated in CLL, while it plays a key role in CLL pathogenesis [49, 50]. Therefore, a potential novel regulatory axis contributing to CLL pathobiology could emerge. However, it is important to clarify that a miRNA and a circRNA should have the same localization to interact, and both RNA types have been detected in the nucleus and cytoplasm based on the literature [51, 52]. Overall, the investigation of potential circRNA/miRNA/mRNA axes would be important since their disruption dramatically affects normal cell function.

Direct visualization using circFISH revealed the intracellular localization of circ‐BAX‐4 and circ‐BCL2L12‐48. More specifically, a distinct subcellular localization was revealed for each circRNA. circ‐BAX‐4, which combines both exonic and intronic sequences, is mainly localized in the nucleus. In contrast, circ‐BAX‐48, which is composed of exons, was detected in both the nucleus and cytoplasm. The current literature supports that circRNAs with intronic regions are localized in the nucleus and can regulate their parental gene expression, which is consistent with the localization of circ‐BAX‐4 [39, 40, 41]. However, to the best of our knowledge, the current studies regarding the exclusively exonic circRNAs support their localization in the cytoplasm. Based on the localization of circ‐BCL2L12‐48 in the nucleus and on fact that the localization of circRNAs is pivotal with regard to their exerted function(s), further investigation is required.

Regarding the expression of the novel circRNAs in the PBMCs of CLL patients and non‐leukemic blood donors, we observed varying expression patterns. Although some of the circRNAs identified in the CLL cell line were detected in the PBMC samples as well, others emerged too. Interestingly, our results reveal that both *BAX* and *BCL2L12* circRNA expression patterns are more complex in CLL patients, compared to those in non‐leukemic blood donors. This finding suggests that the novel circRNAs may also be involved in the pathogenesis of CLL. However, as a future perspective, the sequencing approach applied to the EHEB cell line should be applied to purified B‐cells from CLL patients as well, in order to determine the circRNA expression profile and subsequently explore their functional role and potential contribution to the pathogenesis of CLL.

Undoubtedly, the current study has some limitations. Firstly, we cannot have solid conclusions regarding the expression levels of the novel circRNAs due to the nested‐PCR–based amplification before sequencing library construction. Moreover, the potential role of these molecules has only been investigated *in silico*. The lack of functional experiments from our study limits the understanding of the regulatory effects of the discovered circRNAs. Thus, our future goals include the elucidation of the molecular mechanisms underlying the formation and function of these circRNAs, in an attempt to provide a more comprehensive understanding of their role in CLL. Overall, the present study is one of the few studies having investigated circRNAs in CLL and the first one to explore the different circRNAs produced from apoptosis‐related genes in CLL in such a depth. The experimental workflow led to the discovery of several novel *BAX* and *BCL2L12* circRNAs comprising distinct combinations of exonic and/or intronic sequences; it would be of great interest for these novel circular transcripts to be further investigated in this hematological malignancy.

## Conflict of interest

The authors declare no conflict of interest.

### Peer review

The peer review history for this article is available at https://www.webofscience.com/api/gateway/wos/peer‐review/10.1002/2211‐5463.13672.

## Author contributions

CKK conceived the study, designed experiments, had the supervision, drafted part of the manuscript, produced figures, and critically reviewed the manuscript. PK and PIA performed experiments, collected and analyzed data, drafted the manuscript, and produced figures. AA and AD performed experiments and produced figures. NPM analyzed data. DCS critically reviewed the manuscript. VP and AS provided resources. MB designed experiments, provided resources, supervised part of the work, and critically reviewed the manuscript. SGP provided resources and critically reviewed the manuscript. All authors have read and approved the final version of this manuscript.

## Supporting information


**Fig. S1.** Mean coverage of *BAX* (A) and *BCL2L12* (B) exons and introns by circular and linear transcripts.

**Table S1.** First‐ and second‐round PCR primer pairs, used to generate the amplicons for the nanopore sequencing libraries.

**Table S2.** The primers that were used to amplify and sequence two regions of circ‐BAX‐4 and circ‐BCL2L12‐48, both spanning the back‐splice junction of each circRNA.

**Table S3.** The sequences of the probes that were used to visualize circ‐BAX‐4 and circ‐BCL2L12‐48 with circFISH.

**Table S4.** The primers that were used for the expression analysis of *BAX* and *BCL2L12* circRNAs in CLL and normal PBMC samples.

**Table S5.** The predicted interactions of *BAX* and *BCL2L12* circRNAs interactions with miRNAs.

## Data Availability

The raw nanopore sequencing reads have been deposited to the Sequence Read Archive (SRA) of NCBI, with BioProject accession numbers PRJNA909056 and PRJNA909060.
