# Supplementary material for: Novel circular RNAs of the apoptosis‐related BAX and BCL2L12 genes identified in a chronic lymphocytic leukemia cell line using nanopore sequencing
Source: FEBS Open Bio. 2023 Sep 19;13(10):1953–66. doi: 10.1002/2211-5463.13672 (PMC10549219; doi:10.1002/2211-5463.13672)
Supplement: Supplementary file 1 — Fig. S1. Mean coverage of BAX (A) and BCL2L12 (B) exons and introns by circular and linear transcripts. [file FEB4-13-1953-s003.docx]

**Fig. S1.** Mean coverage of *BAX* (A) and *BCL2L12* (B) exons and introns by circular and linear transcripts.
